# AI-Enabled Predictive Maintenance Framework for Autonomous Mobile Cleaning Robots

**DOI:** 10.3390/s22010013

**Published:** 2021-12-21

**Authors:** Sathian Pookkuttath, Mohan Rajesh Elara, Vinu Sivanantham, Balakrishnan Ramalingam

**Affiliations:** Engineering Product Development Pillar, Singapore University of Technology and Design (SUTD), Singapore 487372, Singapore; sathian_pookkuttath@mymail.sutd.edu.sg (S.P.); rajeshelara@sutd.edu.sg (M.R.E.); vinu_sivanantham@sutd.edu.sg (V.S.)

**Keywords:** artificial intelligence, mobile cleaning robot, vibration source classification, predictive maintenance, deep learning, 1D CNN

## Abstract

Vibration is an indicator of performance degradation or operational safety issues of mobile cleaning robots. Therefore, predicting the source of vibration at an early stage will help to avoid functional losses and hazardous operational environments. This work presents an artificial intelligence (AI)-enabled predictive maintenance framework for mobile cleaning robots to identify performance degradation and operational safety issues through vibration signals. A four-layer 1D CNN framework was developed and trained with a vibration signals dataset generated from the in-house developed autonomous steam mopping robot ‘Snail’ with different health conditions and hazardous operational environments. The vibration signals were collected using an IMU sensor and categorized into five classes: normal operational vibration, hazardous terrain induced vibration, collision-induced vibration, loose assembly induced vibration, and structure imbalanced vibration signals. The performance of the trained predictive maintenance framework was evaluated with various real-time field trials with statistical measurement metrics. The experiment results indicate that our proposed predictive maintenance framework has accurately predicted the performance degradation and operational safety issues by analyzing the vibration signal patterns raised from the cleaning robot on different test scenarios. Finally, a predictive maintenance map was generated by fusing the vibration signal class on the cartographer SLAM algorithm-generated 2D environment map.

## 1. Introduction

Mobile cleaning robots with various capacities are ubiquitous today, for instance in food courts, hypermarkets, hospitals, industries, airports, and homes, and are used for vacuuming, mopping, and sanitizing the environment. Market studies show that the personal and professional mobile cleaning robot growth is expected to reach 24 billion USD by 2026 [1]. However, proper maintenance and deployment in a robot-friendly workspace are crucial for autonomous mobile cleaning robots to avoid malfunction, catastrophic failure, and environmental-related safety issues, including customer dissatisfaction. Currently, manual supervision is widely used to monitor professional cleaning robots’ performance degradation and safety-related issues. However, it is time-consuming, labor and skill-set-dependent, and challenging to deploy due to the lack of historical failure data, especially for the newly developed advanced cleaning robots. Moreover, this periodical manual approach may trigger other issues such as extended downtime, the under-utilization of components, safety issues due to abrupt failure, and high operational and maintenance costs.

Automated predictive maintenance strategies overcome these pitfalls. They are widely used in industrial robots and autonomous vehicles for continuous health monitoring, performance degradation prediction, hazardous operational environment identification, and safety system failure indication. Various methods and techniques were proposed in the literature to implement automated predictive maintenance. A fuzzy inference approach is used in [2] to predict battery power status of robotics systems, and a non-intrusive methodology using torque sensor data for monitoring industrial robot joints in [3]. Similarly, a programmable motion-fault detection method for collaborative robots in [4], a framework to assess the future dynamic behavior and the remaining useful life of industrial robots in [5], and a data-driven predictive maintenance methodology using time-series electrical power data is used to detect manipulator errors in [6].

In recent years, artificial intelligence (AI) powered predictive maintenance (PdM) has been widely studied for automated PdM design. It adopts Machine Learning (ML) and Deep Learning (DL) algorithms for fault detection and classification. These works include a K-means clustering algorithm-based PdM for wafer transfer robot to avoid unplanned downtime proposed in [7], an automatic ML tool based health monitoring system to predict safe stops in a collaborative robot in [8], an Artificial Neural Network (ANN) model to predict the system failure of a packaging robot in [9], an ML-based PdM to detect drive belt looseness in a Cartesian robot in [10], and a DL-based fault diagnosis of industrial robots in [11] using multi-sensor fusion technology. Similarly, an ML-based fault diagnosis for the vehicle brake system is studied in [12] using wavelet applications, a terrain classification study for the autonomous ground vehicles is conducted in [13] adopting a probabilistic neural network, a hierarchical component-based diagnosis and prognosis system proposed for autonomous vehicles in [14] using a Dynamic Bayesian Network (DBN) model, and a DL-model developed to forecast the health of multi-sensor autonomous vehicles by training health index networks in [15].

Though several works are available for industrial robots and autonomous vehicle applications, predictive maintenance of autonomous mobile cleaning robots is not widely studied yet. The PdM system is a mandate function to autonomous mobile cleaning robots to deliver a safe and efficient service when operating in a complex and dynamic change environment, identify any performance degradation, and avoid operational safety issues. Generally, vibration is a key indicator for industrial robots and autonomous vehicles to predict performance degradation and hazardous operating environment identification, which is applicable for autonomous mobile cleaning robot platforms.

This work proposes an AI-powered predictive maintenance framework using vibration as a source for autonomous mobile cleaning robots. A one-dimensional Convolutional Neural Network- (1D CNN) based vibration source classification model was developed and trained to classify the vibration signals arising from mobile cleaning robots. An in-house developed autonomous steam mopping robot–‘Snail’ is used to test and validate the proposed framework with different health conditions and hazardous operational environments.

The rest of this paper is organized as follows: Section 2 explains the literature survey on various vibration signal-based PdM works. Section 3 states the problem definition and motivation of this work. Section 4 gives an overview of the proposed system. Section 5 presents various experiments conducted and the results. Finally, Section 6 concludes the summary of the works.

## 2. Literature Survey

Currently, advanced industries use vibration-based health monitoring systems to detect early signs of the failure of machines and industrial robots. The vibration signals, measured using various vibration measuring sensors like piezoelectric or micro-electro-mechanical systems (MEMS) accelerometers, contain the health information of the equipment. In the literature, many reviews and research works have been conducted on vibration signal-based PdM using DL techniques, primarily for machine components, and industrial robots. The 1D CNN based vibration signal analysis studies for PdM includs a survey on 1D CNN benefits and applications in [16], fault diagnosis of machine components in [17,18,19], structural health monitoring in [20,21,22], and real-time fault diagnosis for power assets in [23]. Toh and Park reported the impact of vibration responses for early structural health monitoring in [24]. The study uses different DL architectures for vibration analysis. Pham et al. applied CNN for fault diagnosis of bearings operated under different shaft speeds in [25]. They represented vibration signals as spectrograms to classify faults with high accuracy. Kolar et al. in [26] proposed a vibration signal-based fault diagnosis framework using the CNN algorithm. The authors used a three-axis accelerometer-generated vibration signal to detect and classify the faults. In [27], Chen et al. evaluated three deep neural networks models, including Deep Boltzmann Machines (DBM), Deep Belief Networks (DBN), and Stacked Auto-Encoders (SAE), to detect rolling bearing faults using vibration signals. Chen and Lee discussed in [28] a DL approach for vibration signal analysis for machining applications. Their study covers the optimisation method for CNN, 1D CNN, and 2D CNN structures with different types of inputs such as raw signal data and time-frequency spectra images. A DL model was developed by Luo et al. to detect early faults of a CNC machine in [29]. The authors used impulse responses from the vibration signals to detect the early mechanical fault under time-varying conditions. A fault diagnosis for the industrial robot was proposed by Wu et al. in [30]. Here, the authors combined three algorithms, including Manifold learning, Treelet Transform, and Naive Bayes, to detect the fault in industrial robots.

The studies mentioned above show that vibration signals are the decisive analyzing element, and DL-based techniques are suitable for the feature extraction from vibration signals for predicting a system’s performance degradation. However, most of the works available are focused on finding a specific fault, severity, or its remaining useful life, and do not consider the external factors for degradation. Moreover, the works are intended mainly for various machine components and industrial robots only. Hence, there is a research gap for monitoring autonomous cleaning robots’ health status and identifying hazardous environmental factors.

## 3. Problem Definition

A properly designed and developed cleaning robot works as required in its planned work environment without abnormal vibration. However, due to continuous operation or the impact of various internal and external environmental factors, the robot performance degrades and generates abnormal vibration signals. In most indoor cleaning robots, external terrain factors such as rough pebble pathways or tactile pavers produce high amplitude vibrations that cause performance degradation issues, such as assembly looseness, sensor misalignment, and faster component deterioration, for example. Vibration due to collisions with walls and other obstacles is an indicator of hazardous operation. It may arise due to the failure or malfunction of obstacle avoidance sensors or the misalignment of safety sensors, or the absence of hazardous object registration in the robot navigation map, such as tiny objects and glass walls (LiDAR sensor is sometimes not accurate in detecting tiny objects below object detection range or glass walls in [31,32,33]). A structural imbalance vibration signal is another indicator for both performance degradation and a hazardous operational environment. It may arise due to wheel damage, wear, a loose assembly of heavier components (battery, water tank), or a robot operating in a poor ground clearance area. Hence, identifying the vibration signal source is mandated to predict the cause of failure or performance degradation and identify the operational safety issues. It will enhance the predictive maintenance actions, for instance, isolating the potential hazardous region quickly, determining the severity and fixing the issues, redirecting to its intended workspace, securing a robot-friendly environment, and includes design improvement planning.

## 4. Overview of the Proposed System

**Figure 1:**
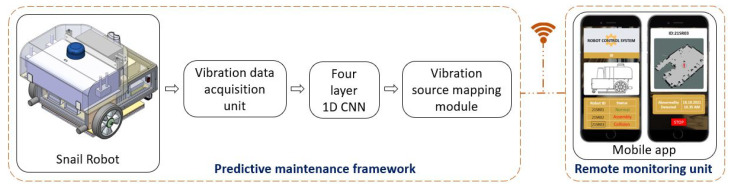
Overview of the proposed DL-based PdM framework.

Figure 1 shows an overview of the AI-enabled PdM framework for an autonomous cleaning robots platform. It uses vibration signals to predict cleaning robot performance degradation and any hazardous operational environment. The overview of the framework is explained as follows. This involves the robot platform details used for tests, the vibration data acquisition unit, the four-layer 1D CNN algorithm, the vibration source mapping module, and the remote monitoring unit.

### 4.1. Autonomous Steam Mopping Robot ‘Snail’

**Figure 2:**
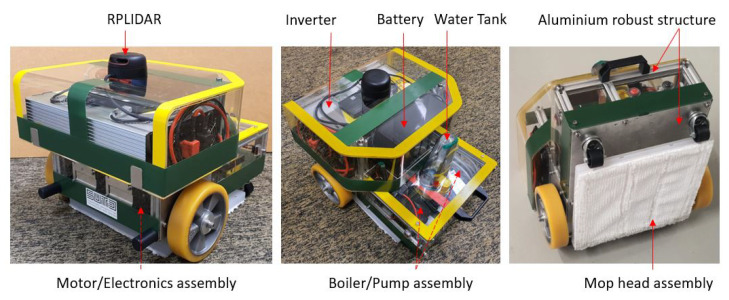
Autonomous steam mopping robot ‘Snail’.

An in-house autonomous steam mopping robot (Snail) designed for cleaning and disinfection of indoor floors is used in this case study, as shown in Figure 2. The overall size of the robot is 40 × 40 × 38 cm, and the total weight is 18 Kg, including inverter, battery, steam boiler, and mop head assembly. An NVIDIA Jetson AGX single-board computer is used to steer the entire operation of the robot, including autonomous navigation, executing predictive maintenance framework, and controlling all other sensors. In addition, the RPLIDAR A2 scanner is used for localization and mapping of the environment, and the Inertial Measurement Unit (IMU) Vectornav VN-100 sensor is used to estimate the motion, orientation, and heading angles of the robot. The robot locomotion was accomplished by a differential wheel drive mechanism with two supporting caster wheels. A D-Link 4G/LTE mobile router is used to control the robot remotely and monitor the robot’s health.

### 4.2. Vibration Data Acquisition Unit

**Figure 3:**
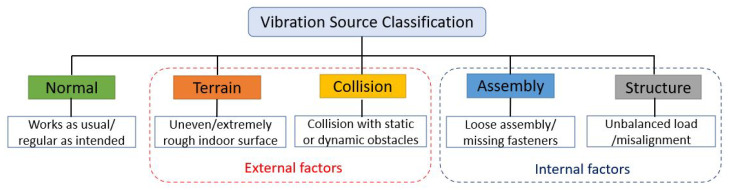
Vibration source classification—Normal and Potential source of failure.

In our study, five vibration classes are used as critical indicators for mobile cleaning robots’ performance degradation and operational safety issues. It is classified under three categories: normal, external factors, and internal factors, as shown in Figure 3. Here, terrain and collision-induced vibration belong to external factors, while assembly and structure-induced vibrations are due to internal factors.

**Figure 4:**
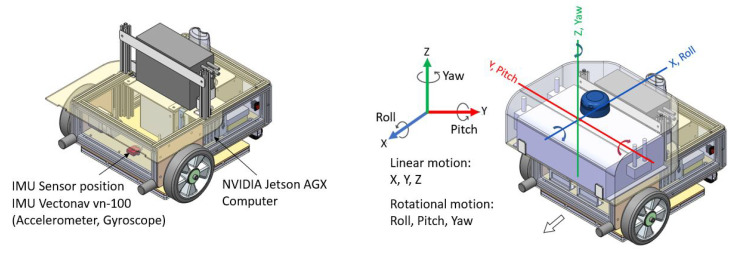
Data acquisition system and Linear-rotational motion of the Snail robot.

The linear and angular motion of the robot will be affected due to internal or external causes of vibration. Hence, we measured the linear acceleration (X, Y, and Z-axis) and angular velocity (roll, pitch, and yaw) of the robot using the onboard IMU sensor (Vectornav VN-100), which reflects the vibration level of the robot. Our IMU data subscription includes angular acceleration calculated from each instance’s current and previous angular velocity. Hence, the robot measures three signal data (linear acceleration, angular velocity, and angular acceleration) in three axes. This total of nine-sensor data collected during the exposure of five vibration source classes was used as the vibration signal data (feature values). The IMU subscription rate was set at 40 Hz, and each sample is grouped into 128 (time steps) data elements, which is every 3.2 s. The collected data is converted into a three-dimensional input array comprising samples, time steps, and features values. Figure 4 shows the data acquisition unit details, mainly the sensor position on the chassis at the center of the differential wheel drive axis.

### 4.3. 1D Convolutional Neural Network (1D CNN)

A 1D CNN model is adopted to build the proposed vibration source classification framework. The 1D CNN is formulated by convolution operations on data vectors as explained in [23]. It consists of signal input data vector *x* (length *N*), a filter vector ω (length *L*), a bias term *b* to best fit for given data, and a nonlinear activation function. The input vector is convolved with the filter vector, and its output layer is represented as in Equation (1). This representation of output layer **c**, of length (N−L+1) is without zero padding. To reduce the number of parameters and highlight the key feature, a max pooling output vector **d** is defined, after each convolution layer, with a kernel size m×1, window function *u*, and filter moving stride *s* as in Equation (2).

(1) $$c\left(j\right)=f\left(\sum_{i=0}^{L-1}\omega \left(i\right)x\left(j-i\right)+b\right),\;j=0,\;1,\;\ldots \;,\;N-1$$

(2) $$d=max\left(u(m\times 1,s)c\right)$$

**Figure 5:**
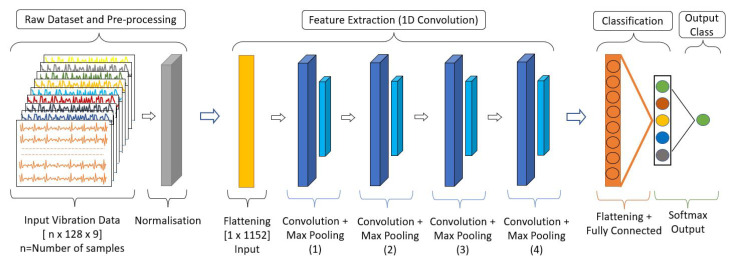
1D CNN Structure.

Figure 5 shows the structure of the predictive maintenance 1D CNN framework. It involves mainly an input layer, four convolutional layers, a Fully Connected (FC) layer, and an output layer. The raw sensor data of the 3D array [n × 128 × 9] gets normalised first. Then each sample is flattened to a 1D array [1 × 1152] for feeding into the CNN. The first two CNN layers use 64 filters and follow kernel size (convolution window) 3. The following two CNN layers use 32 filters and apply the same kernel size. A Rectified Linear Activation Unit (ReLU) is applied to each convolutional layer to learn complex nonlinear patterns in the signal data. After each convolutional layer, max pooling with a stride size of 2 and a dropout layer (dropout rate 0.2) is applied to reduce the feature map dimension and avoid over-fitting. Finally, a flattening function is used to convert the pooled feature map into a 1D array and pass it into the FC layer. A softmax layer is added as the final activation function in the output layer that predicts the multinomial probability. The output layer contains five neurons for the five vibration classes. Table 1 shows the details of the 1D CNN architecture.

### 4.4. Vibration Source Mapping Module

The mapping module performs 2D mapping of the environment using the onboard RPLidar and fuses the CNN predicted vibration source class on the 2D map generated. The Cartographer SLAM (Simultaneous Localisation and Mapping) algorithm is used to generate the 2D environment. It builds a grid-based map for the given environment. The prediction algorithm-generated vibration source classes are fused continuously into this grid map to generate a predictive maintenance map (PdM map). The user or maintenance team can visualize the type of performance degradation and safety-related issues on deployment space through the PdM map.

### 4.5. Remote Monitoring Unit

A smartphone app is developed to visualize the Snail robot’s real-time prediction results for remote health monitoring and control the robot in teleoperation mode, as shown in the overview layout Figure 1. The app is connected through the robot using the MQTT messaging protocol and collects the predicted information in request-based or continuous mode.

## 5. Experiments and Results

This section describes the experimental methods and results. The experiments were performed in four phases: dataset preparation, training the predictive maintenance CNN framework, validating the trained model with test dataset, and real-time field trials.

### 5.1. Data-Set Preparation and Pre-Processing

**Figure 6:**
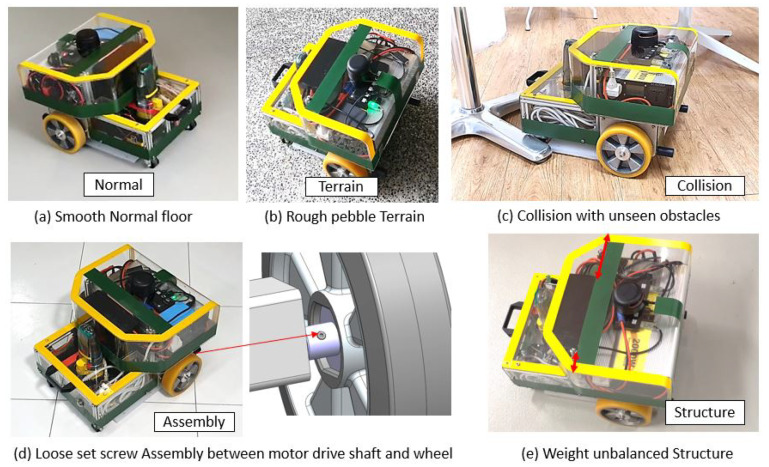
Robot test set up for vibration data collection of five classes.

The dataset preparation involves collecting the vibration signals from the robot with different health states deployed on varying surface conditions, operational speeds, and cleaning patterns. Figure 6 shows the robot test set up for collecting the five classes of vibration including normal operational vibration, rough terrain induced vibration, collision-induced vibration, loose assembly induced vibration, and structure imbalance vibration.

Here, the normal operational and rough surface-induced vibrations were collected by deploying the robot over smooth indoor floors and rough terrain, respectively. Collision-induced vibrations were collected by hitting the robot on different obstacles, including walls and other static and dynamic (human) objects in the environment. The loose assembly-induced vibrations were collected by loosening the robot’s components, such as wheel coupling and mounting brackets. Finally, the unbalanced load-induced (structure) vibration data has been collected by using damaged/worn out wheels, asymmetrically placing heavier components, and operating the robot in a poor ground clearance area. The above mentioned five vibration classes were collected under different surface conditions (tile, concrete, carpet, wooden, vinyl, small and medium-size pebble pathways, and tactile pavers), operational speeds (linear 0.02–0.4 m/s and angular 0.3–1.3 rad/s), and cleaning patterns (straight, zig-zag, and spiral).

**Figure 7:**
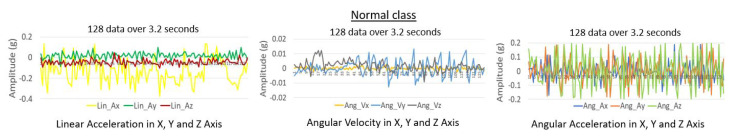
Vibration signals—Normal class.

**Figure 8:**
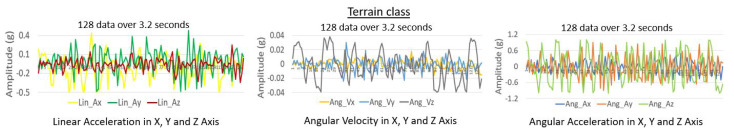
Vibration signals—Terrain class.

**Figure 9:**
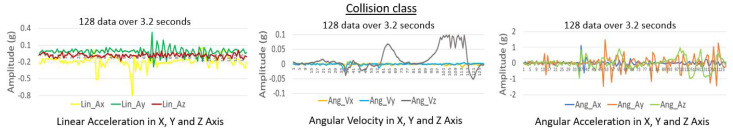
Vibration signals—Collision class.

**Figure 10:**
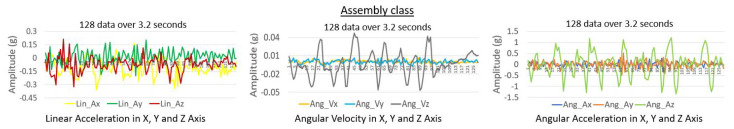
Vibration signals—Assembly class.

**Figure 11:**
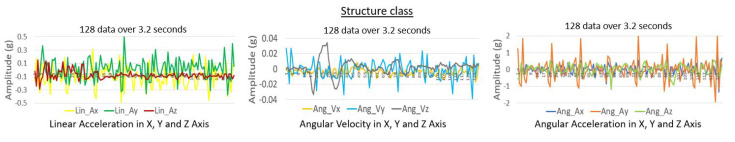
Vibration signals—Structure class.

The Figure 7, Figure 8, Figure 9, Figure 10 and Figure 11 shows the time-amplitude graph for the vibration signals raw data collected for all the five classes across each signal type (linear acceleration, angular velocity, angular acceleration) and its three-axis. The graphs provide a visual representation of how the signals vary through different vibration source classes. It is plotted for one sample (128 data), i.e., captured in 3.2 s.

Data normalisation is applied in the pre-processing stage. The normalisation process involves bringing the raw data into a standard scale without losing information. In our case, the collected data *x* of all the nine features of each class is normalised into −1 to +1 using the Equation (3). Then, the pre-processed dataset is split into training, validation, and test data sets. The training and validation datasets are used to train the model, and the test dataset is used to evaluate the model after training. A total of 2500 samples for each class were recorded and split into 80% for training and 20% for validation. Furthermore, for evaluating the model, a total of 500 samples were collected for each class as test data sets.

(3) $$x_{Normalised}=2\frac{x-min(x)}{max(x)-min(x)}-1$$

### 5.2. Training and Validation

A supervised learning strategy was used in this PdM framework using our unique dataset. Tensorflow [34] deep learning library was used to develop the predictive maintenance CNN framework, and Nvidia GeForce GTX 1080 Ti-powered workstation was used to train the model with the collected dataset. Table 2 shows the hyperparameter settings for training the model. Momentum with gradient descent was used as the optimising strategy to speed up learning and not get stuck with local minima. Adam optimiser (adaptive moment optimization) [35] with three parameters showed better training results: a learning rate of 0.001, the exponential decay rate for the first moment of 0.9, and for the second moment of 0.999. Different epochs were used for testing, and better accuracy was found with 100 epochs and a batch size of 32. The categorical cross-entropy loss function is used while compiling the model to reduce the loss during training and to improve prediction probability.

In the training phase, a K-fold (in our study, K = 5) cross-validation technique is used to evaluate the dataset’s quality, improve generalization, avoid over-learning, and choose the best model for this application. In k-fold cross-validation, the datasets are split into k subsets and k-1 subsets to train the model. The remaining one is for evaluating the model’s performance.

### 5.3. Prediction with Test Dataset

The vibration class prediction efficiency of the trained model is evaluated with the test dataset. A total of 500 test samples were used for each class to evaluate the model. These test datasets have not been used in the training and cross-validation of the model. Accuracy, precision, recall, and F1 Score (Equations (4)–(7)) statistical measure metrics [36] were used to assess the model performance. Here, TP,FP,TN,FN represents the true positives, false positives, true negatives, and false negatives, respectively, as per the standard confusion matrix.

(4) $$Accuracy=\frac{TP+TN}{TP+FP+TN+FN}$$

(5) $$Precision=\frac{TP}{TP+FP}$$

(6) $$Recall=\frac{TP}{TP+FN}$$

(7) $$F1Score=\frac{2\times Precision\times Recall}{Precision+Recall}$$

Table 3 shows the statistical measure result for the test dataset. Accordingly, the model classifies the normal operational vibration class with 89% accuracy, and the hazardous terrain-induced vibration and collision-induced vibration accuracy were predicted with 95% and 92% accuracy, respectively. Furthermore, loose assembly-induced vibration and structure imbalance vibration classes were predicted with 94% and 91% accuracy. The model’s overall classification accuracy (average of five classes) is 92.2%. The above analysis shows that the trained model has accurately classified the five vibration classes collected from the mobile cleaning robot run on different surfaces. Hence, this proposed model is suitable for real-time deployment in mobile cleaning robots for performance degradation and hazardous operational region prediction.

### 5.4. Comparison with Other Algorithms

The performance of the proposed predictive maintenance 1D CNN framework was compared with other commonly used ML/DL classifier models, such as Support Vector Machine (SVM) [37], Multilayer Perceptron (MLP) [38], Long Short-Term Memory (LSTM) [39], and CNN-LSTM [40]. The same training and test dataset, processing resources, and conditions used for the 1D CNN model have been applied for a fair comparison. The CNN-LSTM, LSTM, and MLP models were trained using the TensorFlow library and SVM with Scikit-learn [41] package. The key parameter settings such as optimiser (Adam), learning rate (0.001), and loss function (categorical cross-entropy) for the CNN-LSTM, LSTM, and MLP comparison models were used the same as 1D CNN. For the SVM comparison model, the key parameters ‘C’ and ‘gamma’ values used 100 and 0.01, respectively, and the Radial Basis Function (RBF) kernel was applied. The overall accuracy of each model over five classes and the inference time (millisecond) to process one sample data are given in Table 4.

The comparison results show that our proposed predictive maintenance 1D CNN framework has scored better classification accuracy and took less inference time than the other four algorithms. Hence, it is evident that our proposed system is an optimal algorithm to predict the performance degradation and hazardous operational environment.

### 5.5. Real-Time Prediction

The real-time prediction experiment was tested with the Snail robot in four different environments at the SUTD campus include the lobby, food court, corridor, and lab workspace. These environments were not used for the training data-set collection process. Before the real-time prediction experiment, all the environments were mapped by the cartographer SLAM algorithm using the on-board RPLIDAR A2 for autonomous navigation and predictive maintenance map generation. In our experiment, the continuous field trial was conducted on multiple days to observe the robot performance degradation and hazardous operational prediction. The trained model was configured in robot on-board computer NVIDIA Jetson AGX. Its prediction results (terrain, collision, assembly, or structure-induced vibrations signals) were fused into the cartographer SLAM generated environment map with different colors to identify performance degradation and hazardous operational prediction.

**Figure 12:**
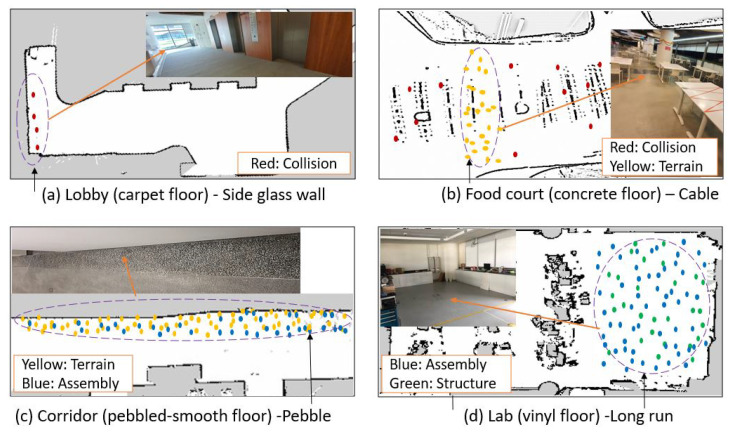
Real time field test case studies.

The first case study was trialed in a lobby environment consisting of a glass sidewall and carpet floor. Here, collision-induced vibrations were registered in the cartographer SLAM algorithm-generated map due to incorrect mapping of the glass wall. The glass wall was covered with a raising curtain in the original map during the mapping time, whereas it was removed while testing. As a result, the robot lost the previously mapped navigation awareness, and RPLidar could not locate the glass as an obstacle. The robot hit on the glass randomly, and the repeated collision-induced vibration marks were captured in the lobby environment 2D map as shown in Figure 12a.

The SUTD food court was our second case study testbed. The food court environment has smooth concrete flooring with dining furniture. The robot trial was conducted during different operational times, including peak hours. Figure 12b shows the SUTD food court set up and its cartographer SLAM map registered with predicted vibration signals. Here, the collision-induced vibration and hazardous surface-induced (terrain) vibrations were recorded in the environment map. Specifically, collision-induced vibration was registered primarily during peak business hours, which arises due to accidental collision with humans, undetected furniture legs, and changes in the position of the dining furniture. Similarly, tactile pavers and cables set on the floor caused hazardous terrain-induced vibrations.

Case study three was conducted in a corridor environment with mixed style flooring with a smooth concrete floor, and a pebble paved rough surface. During our experiment, the robot was first deployed to clean the smooth concrete floor. Here, no abnormal vibrations were reported in the 2D environment map. Later, when the robot started cleaning rough pebbled surfaces, hazardous terrain-induced vibrations marks were seen on the map. When the robot was deployed for a ten day trial on the pebbled surface, a mix of terrain and loose assembly-induced vibrations were registered, as shown in Figure 12c. This is mainly due to the loose assembly of mechanical systems such as loosening of mounting brackets screws and deterioration of mop cloth.

The fourth case study was performed in our SUTD ROAR lab workspace, where the environment filled dynamic objects and the terrain consisted of vinyl flooring. The experiment lasted for four weeks, and its cumulative results in the lab environment were observed. In the first three weeks, the map showed regular drive without capturing any abnormal vibration signature. However, the fourth-week map showed a loose assembly pattern observed from the wobbled wheels due to the loosening of the wheel coupling set screws. The test continued in this wheel-wobbled state and noticeable imbalanced structural vibrations arose due to the battery becoming detached from its bracket and creating an unbalanced weight distribution. Figure 12d shows the four-week test results for case study four, where the environment map depicts loose assembly and unbalanced weight structural patterns.

As per the experimental results, we observed that one source of failure might lead to another if no action is taken. This way, if several sources of failure are present, the model will predict the predominant failure of the robot in that particular instance. Hence, through the mobile app, the maintenance person can remotely stop the robot as soon as the initial abnormal class is registered, avoiding chances for multiple failures or hazards to the environment.

Table 5 shows the statistical measure result for 100 test samples collected from the real-time case study. Here, the sample data were collected from the Snail robot through the mobile app on request-based sample mode and performed the statistical measure using the confusion matrix function. The table results indicate that the algorithm classified the five different vibration sources with an average accuracy of 88.9%, 93.5%, 91.8%, 92.1%, and 88.7% for normal, terrain, collision, assembly, and structure, respectively. In contrast with offline results, the model average prediction accuracy of five classes in real-time tests is slightly less (91%) due to external noise and various sampling periods. However, it can be reduced by fussing multiple IMU sensors or adding a noise cancellation function in the preprocessing stage.

## 6. Conclusions

An AI-enabled predictive maintenance framework was proposed for mobile cleaning robots to monitor performance degradation and identify operational safety issues. The proposed framework was tested and validated with our in-house developed autonomous steam mopping robot ‘Snail’. A four-layer 1D CNN model was developed using TensorFlow API and trained with five vibration signal datasets generated from the Snail robot with different health conditions. The efficiency of the proposed predictive maintenance framework was evaluated with offline and real-time field tests. The experimental results show that the model scored 92.2% accuracy for classifying the performance degraded and hazardous operational vibration signals in offline tests and took 0.162 ms to process one test sample. In the real-time field test, the algorithm accurately predicted robot performance degradation and operational safety issues with an accuracy of 91%. The predicated vibration signal class was fused into the cartographer SLAM-generated environment map to track the performance degradation and identify the operational safety issues. This will help manufacturers and cleaning maintenance companies to choose the right maintenance strategy, rental policy, or improve the robot design and assembly.

## Figures and Tables

**Table 1:** 1D CNN Architecture.

Layer	Kernel Size	Stride	Filters	Data Shape
Input				(1152, 1)
Conv. 1D-1	3 × 1	1	64	(1150, 64)
Max Pool 1D-1	2 × 1	2 × 1		(575, 64)
Conv. 1D-2	3 × 1	1	64	(573, 64)
Max Pool 1D-2	2 × 1	2 × 1		(287, 64)
Conv. 1D-3	3 × 1	1	32	(285, 32)
Max Pool 1D-3	2 × 1	2 × 1		(143, 32)
Conv. 1D-4	3 × 1	1	32	(141, 32)
Max Pool 1D-4	2 × 1	2 × 1		(71, 32)
Fully Connected				(100)
Output (Softmax)				(5)

**Table 2:** HyperParameters setting.

Parameter	Values/Function
Optimizer	Adam
Learning rate	0.001
Batch Size	32
Epochs	100

**Table 3:** Offline test result.

Vibration Source	Precision	Recall	F1 Score	Accuracy
Normal	0.86	0.90	0.89	0.89
Terrain	0.97	0.95	0.96	0.95
Collision	0.92	0.92	0.92	0.92
Assembly	0.93	0.94	0.94	0.94
Structure	0.86	0.92	0.93	0.91

**Table 4:** Accuracy comparison with other models.

Model	Accuracy (%)	Inference Time (ms)
1D CNN	92.2	0.162
CNN-LSTM	88.1	0.258
LSTM	85.4	0.276
MLP	79.8	0.193
SVM	77.5	1.675

**Table 5:** Real-time prediction accuracy of five classes.

Vibration Source	Normal	Terrain	Collision	Assembly	Structure
**Prediction (%)**	88.9	93.5	91.8	92.1	88.7
